# Improved cycling performance with ingestion of hydrolyzed marine protein depends on performance level

**DOI:** 10.1186/1550-2783-9-14

**Published:** 2012-04-10

**Authors:** Geir Vegge, Bent R Rønnestad, Stian Ellefsen

**Affiliations:** 1 The Lillehammer Research Center for Medicine and Exercise Physiology, Lillehammer University College, Lillehammer, Norway; 2 Lillehammer University College, Lillehammer, P.O.Box 952 N-2604, Norway

**Keywords:** Hydrolyzed protein, Sport nutrition, Cycling performance

## Abstract

**Background:**

The effect on performance of protein ingestion during or after exercise is not clear. This has largely been attributed to the utilization of different scientific protocols and the neglection of accounting for factors such as differences in physical and chemical properties of protein supplements and differences in athletic performance level.

**Methods:**

We hypothesized that ingestion of unprocessed whey protein (15.3 g·h^-1^) together with carbohydrate (60 g·h^-1^), would provide no ergogenic effect on 5-min mean-power performance following 120 min cycling at 50% of maximal aerobic power (2.8 ± 0.2 W·kg^-1^, corresponding to 60 ± 4% of VO_2max_), compared to CHO alone (60 g·h^-1^). Conversely, we hypothesized that ingestion of the hydrolyzed marine protein supplement NutriPeptin™ (Np, 2.7 g·h^-1^), a processed protein supplement with potentially beneficial amino acid composition, together with a PROCHO beverage (12.4 g·h^-1 ^and 60 g·h^-1^, respectively) would provide an ergogenic effect on mean-power performance. We also hypothesized that the magnitude of the ergogenic effect of NpPROCHO would be dependent on athletic performance. As for the latter analysis, performance level was defined according to a performance factor, calculated from individual pre values of W_max_, VO_2max _and 5-min mean-power performance, wherein the performance of each subject was ranked relative to the superior cyclist whos performance was set to one. Twelve trained male cyclists (VO_2max _= 65 ± 4 ml·kg^-1^·min^-1^) participated in a randomized double-blinded cross-over study.

**Results and conclusions:**

Overall, no differences were found in 5-min mean-power performance between either of the beverages (CHO 5.4 ± 0.5 W·kg^-1^; PROCHO 5.3 ± 0.5 W·kg^-1^; NpPROCHO 5.4 ± 0.3 W·kg^-1^) (P = 0.29). A negative correlation was found between NpPROCHO mean-power performance and athletic performance level (using CHO-performance as reference; Pearson R = -0.74, P = 0.006). Moreover, ingestion of NpPROCHO resulted in improved 5-min mean-power performance relative to ingestion of CHO in the six lesser performing subjects compared to the six superior performing subjects (P < 0.05). This suggests that with the current protocol, NpPROCHO provided an ergogenic effect on 5-min mean-power performance in athletes with a lower performance level.

## Background

Supplementation of nutrients is generally accepted as having an ergogenic effect on long-term physical performance (> 2 h) [1]. While carbohydrate (CHO) intake seems to be crucial, with current recommendations ranging from 30-70 g·h^-1 ^[1,2], the need for additional nutrients such as protein (PRO) remains elusive. Some studies have suggested that the addition of protein improves performance [3,4], while others have suggested that it has no effect [2,5-7] or even a negative effect [8]. The observed discrepancies have been ascribed factors such as inappropriate choices of test procedures [2,3,6,9], inadequate interpretation of data [9], differences in caloric intake [3] and the physical properties of the protein source [10], and has led to discussion [9,11]. Taken together, available data sets points towards a complex and unresolved causal connection between protein intake and performance level. The complexity is underlined by the meta-analysis by Stearns et al. [3], which suggested that adding protein to isoCHO beverages, thereby increasing the caloric intake, results in improved performance in time-to-exhaustion trials but not in time trial protocols.

Of particular interest as factors that may determine the ergogenic effect of nutrient supplements is the athletic performance level and the chemical structure and composition of the ingested nutrients. As for the former, available studies have investigated the effect of protein ingestion in athletes with a broad spectrum of performance levels, with mean maximal oxygen consumption (VO_2max_) values ranging from 46 to 63 ml·kg^-1^·min^-1^. This suggests extensive individual variation in physiology, which is likely to affect the outcome of such experiments. More specifically, differences in parameters such as genetics, epigenetics and training status are likely to be associated with differences in responses to concurrent ingestion of nutrients and physical activity. This will lower the statistical power of any given experiment and thus challenges straightforward evaluation of groupwise effects and causalities. Indeed, accounting for differences in performance level has been pointed out as a weakness of previous studies in sport nutrition [9]. This is in line with recent publications suggesting that individual variation in physiology has been erroneously ignored as an underlying determinator of sport performance [12-14].

Ingestion of protein supplements that vary in refinement status and chemical structure are likely to have differential effects on physical performance. This remains one of the largely unexploited aspects of sports nutrition and a particularly intriguing is the potentially ergogenic effect of hydrolyzed protein [15]. Indeed, hydrolyzed protein supplements are emerging as commercially available products [15]. Until now, however, the scientific basis for recommending hydrolyzed protein intake during physical activity is limited. Although experiments have suggested a positive effect on late-stage long-term cycling performance [10] and on molecular adaptations to and recovery from resistance training [16,17], no study has compared the effects of protein and hydrolyzed protein on endurance performance. The effects of hydrolyzed protein supplementation remains elusive.

Furthermore, different sources of protein provide protein supplements with different amino acid composition. This will bring about differences in nutrient absorption kinetics and metabolic responses, which surely will affect ergogenic properties. For example, whey protein elicits a different absorption profile than casein protein and also affects whole body protein metabolism in a different way [18]. Amino acid composition can thus be anticipated to have an impact on the ergogenic effects of a protein supplement in much the same way as protein hydrolyzation was hypothesized to have. Intriguingly, compared to ingestion of soy and casein PRO, long-term ingestion of fish protein hydrolysate has been indicated to result in increased fatty acid oxidation in rats [19], an effect that has been linked to a high content of the amino acids taurine and glycine [19,20]. In the context of human sport nutrition, ingestion of fish protein hydrolysate thus emerges as an interesting candidate for improving physical performance, potentially exerting its effect by shifting the metabolism towards fatty acids and thus away from glycogen, delaying the depletion of glycogen stores that typically coincides with physical exhaustion [21,22].

We hypothesized that there would be no ergogenic effect of ingesting a protein + carbohydrate (PROCHO) beverage (15.3 g·h^-1 ^and 60 g·h^-1^, respectively) on 5-min mean-power cycling performance following 120 min of steady-state cycling at moderate intensity (50% of maximal aerobic power, W_max_) in trained cyclists (VO_2max _ranging from 60 to 74 ml·kg^-1^·min^-1^; mean 65 ± 4) compared to ingesting a carohydrate (CHO) beverage (60 g·h^-1^). Conversely, we hypothesized that adding the codfish-based hydrolyzed protein supplement Nutripeptin™ (Np, 2.7 g·h^-1^) (Nutrimarine Innovation AS, Bergen, Norway) to the PROCHO beverage (12.4 g·h^-1 ^and 60 g·h^-1^, respectively) (NpPROCHO) would result in improved performance compared to CHO and PROCHO alone. We further hypothesized that the extent of the ergogenic effect resulting from NpPROCHO ingestion would correlate with athletic performance level measured as a performance factor calculated from W_max_, VO_2max _and familiarization test 5-min mean-power cycling performance.

## Methods

### Subjects

Twelve moderately to well-trained male cyclists, aged 19-27 years (mean 22 ± 2) and VO_2max _60-74 ml·kg^-1^·min^-1 ^(mean 65 ± 4) were recruited by public advertisement. The cyclists were required to having performed a minimum of 6 h of endurance training weekly during the six months leading up to the study, with a main focus on cycling. All cyclists signed an informed consent form prior to participation and the study was approved by the Southern Norway regional division of the National Committees for Research Ethics. Three of the initial 16 cyclists did not make the inclusion requirements of the study and were excluded from data analyses, while a fourth athlete dropped out of the study due to illness.

### Experimental design

VO_2max _was assessed at baseline and 60 ml·kg^-1^·min^-1 ^was set as an inclusion criteria. The effects of ingesting each of the three beverages (CHO, PROCHO and NpPROCHO) on physical performance was tested on three separate test days, separated by at least 4 days and no more than 10 days. The study was designed and carried out in a randomized, double-blinded and crossed-over manner. The three test days consisted of 120 min cycling at 50% of maximal aerobic power (W_max_), as calculated from the VO_2max _data set in accordance with Rønnestad, Hansen and Raastad [23]. For each of the three test days, the 120 min of steady-state cycling was accompanied by ingestion of 180 mL of one of the beverages at 15 min intervals. Four minutes after the 120 min of cycling, a 5-min mean-power performance test was performed.

### Beverages

The CHO beverage contained 8.3% maltodextrin (60 g·h^-1^). The PROCHO beverage contained 2.1% intact whey protein (15.3 g·h^-1^) and 8.3% maltodextrin (60 g·h^-1^). The NpPROCHO beverage contained 0.4% Nutripeptin™ (Np, 2.7 g·h^-1^) (Nutrimarine Innovation AS, Bergen, Norway), 1.7% intact whey protein (12.4 g·h^-1^) and 8.3% maltodextrin (60 g·h^-1^). CHO ingestion was set to a level sufficiently high to ensure maximal CHO uptake at all three test day [1]. Accordingly, the three beverages contained equal amounts of CHO, which is a functional prerequisite for any sport beverage. The two protein-containing beverages were supplied with iso-caloric amounts of protein.

All three beverages were supplemented with the same flavour. The participants still reported the different beverages to have distinct tastes. Importantly, however, the identity of the beverages was not at any time revealed to either the participants or to the test leader. Moreover, because the participants had no previous experience with the beverages and did not know their detailed composition, they could not identify the different beverages. Notably, Np is not a purified protein source, but rather consists of proteolyzed tissue. Compared to for example mixtures of casein protein it contains excessive amounts of B-vitamin complexes. Importantly, B-vitamins does not seem to provide an ergogenic effect on endurance performance in humans [24].

### Test procedure

The cyclists were instructed to refrain from intense exercise for the 48 hours preceding each test. They were also instructed to prepare for each test as if it was a competition event and to prepare for the different test sessions in the same way (i.e. ingesting the same type of meal at a set time interval from the test session). They were restricted from eating food for the 90 min preceding each test and from consuming coffee or other caffeine-containing products for the 4 h preceding each test. The cyclists were cooled with a fan throughout the exercise bouts. All tests were performed under similar environmental conditions (20-22°C). For each cyclist, the three tests involving ingestion of beverages were performed at approximately the same time of day to avoid circadian variance. All cycling tests were performed on the same electromagnetically braked cycle ergometer (Lode Excalibur Sport, Lode B. V., Groningen, the Netherlands), which was adjusted in a standardized manner to each cyclist's preferred seat height, distance between the seat and the handle bars, and horizontal distance between the tip of the seat and the bottom bracket. Cyclists were allowed to choose their preferred cadence during all cycling tests (no differences were found between test days; data not shown) and they were allowed to use their own shoes and pedals.

#### Test of VO_2max _and familiarization to the 5-min mean-power test

In the first test session, the cyclists performed an incremental cycle ergometer test for determination of VO_2max_, as previously described by Ronnestad et al. [23]. The session was preceded by 20 min of low intensity warm-up on the cycle ergometer, in which the last part included two 45 s periods at higher intensities. Before starting the VO_2max _test the cyclists rested for 2 min. The VO_2max _test was initiated with 1-min cycling at a power output corresponding to 3 W·kg^-1 ^(rounded down to the nearest 50 W). Power output was then increased by 25 W every 1 min until exhaustion. When the cyclists evaluated that they could not manage another 25 W increase in power output, they were encouraged to continue cycling at the current power output for as long as possible (usually 30-90 s). Oxygen consumption and respiratory exchange ratio (RER) were measured (30 s sampling time) using a computerized metabolic system with a mixing chamber (Oxycon Pro, Erich Jaeger, Hoechberg, Germany) that was calibrated according to manufacturer's recommendations.

Heart rate (HR) was measured continuously throughout the VO_2max _test using a HR monitor (Polar, Kempele, Finland). Maximal aerobic power (W_max_) was calculated as the mean power output during the last 2 min of the incremental test. W_max _values were utilized to determine power output to be used during the prolonged cycling events on the three test days involving beverage ingestion. After the incremental VO_2max _test, the cyclists performed 15 min of low-intensity cycling before the test session was completed with a 5-min mean-power familiarization test. To ensure stable performance level of the participants during the entire experimental period, the VO_2max _test was repeated 4-10 days after the last test day with beverage ingestion. No differences were found between the first and the last VO_2max _test (65.0 ± 4 vs 65.6 ± 6 ml·kg^-1^·min^-1^; P = 0.79).

#### Prolonged cycling followed by 5-min mean-power cycling

On each of the three test days involving ingestion of beverages, the cyclists performed 120 min of cycling at 207 ± 21 W, representing 50% of W_max_, followed by a 5-min mean-power test. The duration and intensity of the bout of prolonged cycling was based on the pre-exhausting phase used in similar studies [e.g. [6]]. During the prolonged cycling, the ergometer was in a cadence-independent mode (constant Watt-production), so that the pre-set power output was not affected by the cyclist's chosen cadence. Cyclists were allowed to occasionally stand in the pedals during the prolonged cycling, but not during the final 5-min mean-power test.

Four min after completion of 120 min of prolonged cycling the 5-min mean-power test was performed. In line with an earlier study [25,26], the 5-min mean-power test was chosen as a functional measure of the capacity for very intensive cycling, such as occurs during a breakaway attempt, crosswind cycling, or steep uphill cycling, all of which may be decisive situations in a road race. For the 5-min mean-power test, the ergometer mode was changed to cadence-dependent mode, in which the power output increases with increasing cadence according to the formula: W = L × (rpm)^2^, where W is the power output, rpm is the cadence, and L is a constant determining the electronic gearing of the system. L was set to 0.044 based on the prediction that the mean power output during the 5-min mean-power test would be between 360 and 400 W, as suggested from findings in a previous study [25]. All cyclists were encouraged to produce as high a mean power output as possible during the 5-min mean-power test. Towards the end of the 5-min test, all subjects received encouraging feedback on power output production and time elapsed, but not HR or cadence, to ensure maximal performance. The mean power output was calculated and used in statistical analyses.

**Figure 1:**
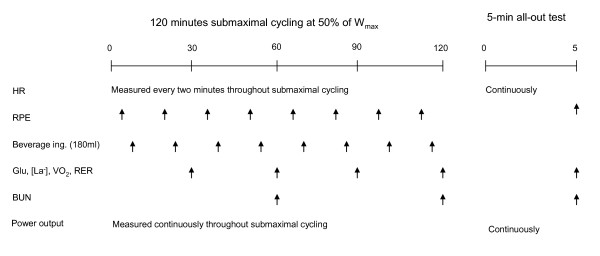
**Schematic presentation of the test protocol**. Metabolic and physiological measures include heart rate (HR), rate of perceived exertion (RPE), oxygen consumption (VO_2_), respiratory exchange ratio (RER), blood glucose (Glu), blood lactate (La-), blood urea nitrogen (BUN) and power output measured as watt (W).

During the 120 min of pre-exhausting exercise, data on HR and cadence were collected every two min and data on the rate of perceived exertion (RPE) was collected every 15 min. Oxygen uptake, CO_2 _production and RER data were collected for 3-min intervals every 30 min. Blood glucose concentration and blood lactate concentration were measured in whole blood from the finger tips using the Contour blood glucose monitoring system (Bayer Healthcare, NY, USA) and the Lactate protein LT-1710 analyzer (Arcray Inc. Kyoto, Japan), respectively. This was done every 15 min. Blood urea nitrogen (BUN) was measured in whole blood from fingertips using an i-STAT^® ^handheld clincial analyzer with EG-8+ cartridges (Abbott Laboratories, Abbott Park, IL, USA) at onset and after completion of the 120 min event. See Figure 1 for a schematic presentation of the data collection process.

During the 5-min mean-power test the following parameters were continuously measured: cadence, HR, VO_2_, CO_2 _production and RER data. Immediately after the 5-min mean-power test, blood lactate was measured in whole blood from the finger tips as previously described and RPE was registered. See Figure 1 for a schematic presentation of the data collection process.

Unfortunately, due to a technical flaw with the equipment for metabolic assessment complete data sets for VO_2 _and RER was only obtained for six of the twelve participants. However, as the main hypothesis was connected to power output data obtained during the 5-min mean-power tests, this was evaluated to be of minor consequences for the outcome of the study.

### Statistics

In general, physiological data from the 120 min of prolonged cycling were analyzed for beverage-specific differences by repeated measures two-way ANOVA (HR, VO_2_, RER, blood lactate, and blood glucose). Within-beverage-test changes were analyzed by a paired *t*-test with a Bonferroni adjustment. BUN-data from the 120 min of prolonged cycling were analyzed for beverage-specific differences and for within-test changes by a paired *t*-test with Bonferroni adjustment. In these calculations, BUN-values at 30, 60, 90 and 120 min were referenced to BUN-values at 0 min which was set to 1.0. Power output data from the 5-min mean-power test were analyzed for beverage-specific differences by a repeated measures one-way ANOVA. Moreover, the linear relationship between beverage-specific 5-min mean-power output performance and pre-test performance level measured as a performance factor, calculated from W_max_, VO_2max _and familiarization test 5-min mean-power cycling performance (see Table 1 for thorough description), was analyzed using Pearson correlation, with subsequent calculation of 95% confidence intervals. In this analysis and in all other analyses relating mean-power cycling performance to performance level, NpPROCHO and PROCHO performance was assessed as performance in percentage of CHO performance. The reason for this is that protein-supplementation was evaluated to be beneficial only if it improves performance compared to CHO-only, which is heavily supported in the literature as a prerequisite for long-term endurance performance [1,2]. Accordingly, NpPROCHO and PROCHO performance was evaluated to be interesting only in light of CHO performance, and CHO performance was set as baseline. Furthermore, in an analysis related to the correlation analysis, the cyclists were divided into two equally sized groups based on their individually calculated performance factor. Subsequent to this, the effect of ingesting NpPROCHO and PROCHO, respectively, relative to CHO was tested between the two groups with a unpaired *t*-test. Furthermore, a comparison of the effect of ingesting NpPROCHO and PROCHO relative to CHO was performed within each performance groups with a paired *t*-test. For this within-group analysis, we also calculated the effect size (ES) (Cohen's *d*). For all analyses, P < 0.05 was considered significant. In analyses involving Bonferroni adjustments, P < 0.017 was considered significant. All statistical calculations were performed using Graphpad Prism5 (GraphPad Software Inc., California, USA). The effect size (ES) calculation was performed using a web resource http://www.uccs.edu/~faculty/lbecker/. All values are mean ± SD, unless otherwise stated.

**Table 1:** Calculation of a performance factor from pretest values of VO_2max_, W_max _and 5-min test mean-power performance for performance-based ranking of the cyclists

Subject	VO_2max_		W·kg^-1 ^5 min test		W_max_		Performance factor
	**raw**	**normalized**	**raw**	**normalized**	**raw**	**normalized**	**average of normalized quantity**

1	62	0.84	4.4	0.75	5.0	0.78	0.79

2	60	0.81	4.8	0.80	4.9	0.76	0.79

3	61	0.83	4.8	0.80	5.1	0.80	0.81

4	63	0.85	4.4	0.74	5.5	0.86	0.82

5	60	0.81	4.9	0.83	5.8	0.91	0.85

6	66	0.89	5.0	0.84	5.7	0.88	0.87

7	64	0.87	5.4	0.92	5.5	0.87	0.88

8	66	0.89	5.3	0.90	5.8	0.91	0.90

9	71	0.96	5.4	0.91	5.4	0.84	0.90

10	67	0.91	5.3	0.89	6.0	0.94	0.91

11	68	0.92	5.9	1.00	6.1	0.95	0.96

12	74	1.00	5.7	0.95	6.4	1.00	0.98

First, for each of the three parameters, the superior performing cyclist was identified value was then utilized for normalization of the performance of the other cyclists, i.e. the performance of each of the other cyclists was calculated as a fraction of the superior performance, thus showing values < 1. For each subject, the ultimate performance factor was calculated as the mean of the normalized VO_2max_, W_max _and 5-min test mean-power performance values.

## Results

### 120 min submaximal exercise

During the prolonged cycling the athletes were exercising at 62 ± 4% of VO_2max_. Ingestion of the three supplements CHO, PROCHO, and NpPROCHO did not provide differences in HR, VO_2_, or RER at 30 min, 60 min, 90 min, or 120 min of the prolonged submaximal cycling (Table 2). Nor did the three beverages result in differences in blood glucose and blood lactate (Table 3) or in RPE (mean values ranging from 11.1 to 13.5 across time points and supplements during the prolonged cycling; data not shown). The supplements did, however, result in differences in the concentration profile of BUN. While ingestion of CHO did not result in changes in BUN levels between baseline (6.3 ± 1.5 mM) and 120 min (6.7 ± 1.8 mM) of steady-state cycling, ingestion of PROCHO and NpPROCHO resulted in changes from 5.9 ± 1.1 mM to 7.7 ± 1.8 mM (P < 0.017) and from 6.1 ± 1.5 to 7.5 ± 1.9 mM (P < 0.0003), respectively (Table 3). The NpPROCHO beverage was associated with higher BUN values after 120 min of cycling than the CHO beverage (P < 0.017), an effect that was not quite found for the PROCHO beverage (P = 0.03) (Table 3). No difference was found between PROCHO and NpPROCHO beverages (P = 0.44).

**Table 2:** Heart rate (HR), oxygen consumption (VO_2_), and respiratory exchange ratio (RER) during 120 min submaximal cycling at 50% of maximal aerobic power with ingestion of either carbohydrate (CHO), protein + carbohydrate (PROCHO) or Nutripeptin™ + protein + carbohydrate (NpPROCHO).

Degree of completion	HR (bpm)			VO2 (ml·kg-1·min-1)			RER		
	
	*CHO*	*PROCHO*	*NpPROCHO*	*CHO*	*PROCHO*	*NpPROCHO*	*CHO*	*PROCHO*	*NpPROCHO*
25%	141 ± 9	141 ± 8	144 ± 7	39.6 ± 3.0	39.7 ± 3.0	40.2 ± 3.4	0.91 ± 0.01	0.92 ± 0.02	0.91 ± 0.02

50%	142 ± 10	144 ± 10	146 ± 7	39.4 ± 3.0	40.1 ± 3.3	40.4 ± 3.9	0.91 ± 0.01	0.92 ± 0.02	0.90 ± 0.01

75%	143 ± 10	146 ± 10	147 ± 8	40.0 ± 3.4	40.4 ± 3.4	41.1 ± 4.2	0.90 ± 0.01	0.91 ± 0.03	0.90 ± 0.01

100%	149 ± 12	150 ± 12	150 ± 9	40.9 ± 3.4	41.3 ± 3.2	41.5 ± 4.8	0.88 ± 0.02	0.90 ± 0.04	0.89 ± 0.01

No differences were found between groups. N = 12 for HR; N = 6 for VO_2 _and RER

**Table 3:** Lactate, blood glucose and Blood Urea Nitrogen (BUN) concentrations in venous blood previous to, during and after 120-min of submaximal cycling at 50% of maximal aerobic power with ingestion of either carbohydrate (CHO), protein + carbohydrate (PROCHO) or Nutripeptin™ + protein + carbohydrate (NpPROCHO).

Degree of completion	Lactate (mmol·L^-1^)			Glucose (mmol·L^-1^)			BUN (mmol·L^-1^)		
	
	*CHO*	*PROCHO*	*NpPROCHO*	*CHO*	*PROCHO*	*NpPROCHO*	*CHO*	*PROCHO*	*NpPROCHO*
0%	1.4 ± 0.3	1.4 ± 0.4	1.5 ± 0.5	5.4 ± 0.6	5.3 ± 0.7	5.3 ± 1.0	6.3 ± 1.5	5.9 ± 1.1	6.1 ± 1.5

25%	1.4 ± 0.4	1.5 ± 0.6	1.6 ± 0.4	5.8 ± 0.6	5.7 ± 0.5*	6.1 ± 1.1*	NA	NA	NA

50%	1.4 ± 0.2	1.3 ± 0.4	1.6 ± 0.4	5.5 ± 0.6	5.3 ± 0.4	5.3 ± 0.6	NA	NA	NA

75%	1.2 ± 0.3	1.2 ± 0.4	1.3 ± 0.2	5.2 ± 0.7	5.4 ± 0.4	5.5 ± 0.7	NA	NA	NA

100%	1.3 ± 0.5	1.3 ± 0.2	1.4 ± 0.5	5.5 ± 0.6	5.6 ± 0.4	5.7 ± 0.5	6.7 ± 1.8^a^	7.7 ± 1.8**, ^ab^	7.5 ± 1.9***, ^b^

Asterixes (*, ** and ***) denote changes in concentrations that occur during the time-course of each particular subset of prolonged cycling (compared to baseline set to 0%). * = P < 0.017, ** = P < 0.003, *** = P < 0.0003. Letters (a and b) denote differences in concentrations that occur between subsets of prolonged cycling. N = 12

### 5-min mean-power test performance

**Figure 2:**
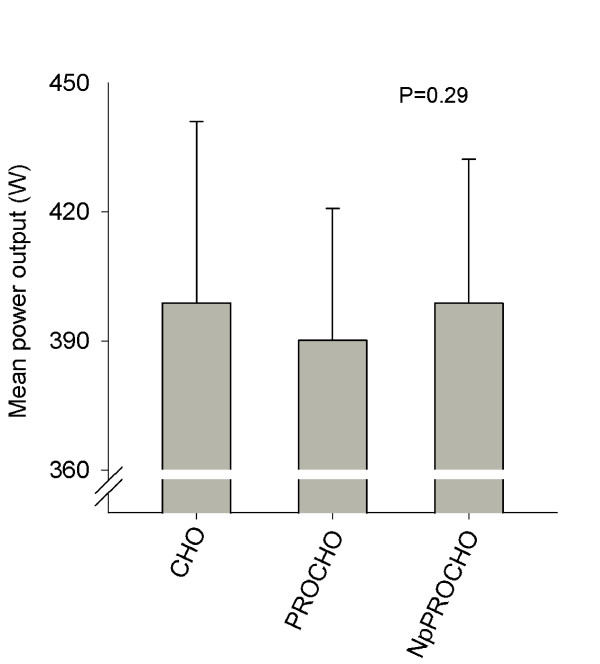
**Mean power output during the 5-min mean-power test following 120-min submaximal cycling at 50% of maximal aerobic power with ingestion of either carbohydrate (CHO), protein + carbohydrate (PROCHO) or Nutripeptin™ + protein + carbohydrate (NpPROCHO)**. No differences were found between beverages. N = 12.

**Figure 3:**
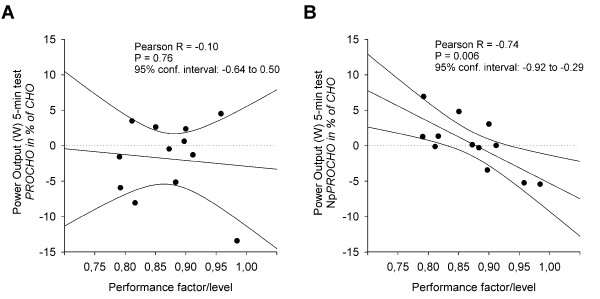
**Correlation between performance level measured as a performance factor calculated from W_max_, VO_2max _and familiarization test 5-min mean-power cycling performance (see Table 2 for thorough description) and performance in 5-min mean-power test following 120 min submaximal cycling at 50% of W_max _with ingestion of either A) protein + carbohydrate (PROCHO) or B) Nutripeptin™ + protein + carbohydrate (NpPROCHO)**. Power-output values for the two beverages were referenced to values obtained for the carbohydrate (CHO) beverage, which was defined as baseline performance. Values on the Y-axis thus depicts the difference in performance between PROCHO and CHO ingestion and NpPROCHO and CHO ingestion, respectively, and is denoted as percentage.

**Figure 4:**
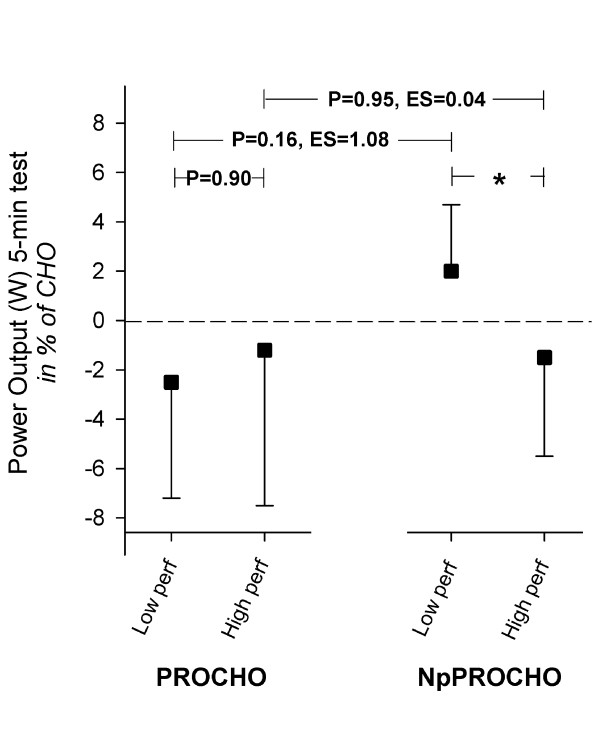
**The effect of ingesting A) protein + carbohydrate (PROCHO) or B) Nutripeptin™ + protein + carbohydrate (NpPROCHO) on performance in a 5-min mean-power test following 120 min submaximal cycling at 50% of W_max _in the six lesser performing cyclists (lesser perf) compared to the six superior performing cyclists (superior perf)**. Power-output values for the two beverages were referenced to values obtained for the carbohydrate (CHO) beverage, which was defined as baseline performance. Values on the Y-axis thus depicts the difference in performance between PROCHO and CHO ingestion and NpPROCHO and CHO ingestion, respectively, and is denoted as percentage. * = P < 0.05. N = 12.

Mean power output during the 5-min mean-power test was not different between beverages; CHO 399 ± 42 W (5.4 ± 0.5 W·kg^-1^), PROCHO 390 ± 31 W (5.3 ± 0.5 W·kg^-1^) and NpPROCHO 399 ± 33 W (5.4 ± 0.3 W·kg^-1^) (P = 0.29, Figure 2). No differences were found in control parameters RPE and blood lactate between beverages as sampled directly after the 5-min mean-power test (data not shown). However, a negative correlation was found between performance in the NpPROCHO 5-min mean-power test and athletic performance level measured as a performance factor, as developed in Table 1 (Pearson R = -0.74 with 95% confidence interval -0.92 to -0.29, P = 0.006, Figure 3), a correlation that was also found between NpPROCHO 5-min mean-power performance and each of the subcomponents of the performance factor (W_max_, Pearson R = -0.74, P = 0.006; VO_2max_, Pearson R = -0.67, P = 0.02 *and *5-min mean-power-output from the familiarization test, Pearson R = -0.66, P = 0.02). No such correlation was found for the PROCHO beverage (Figure 3). The NpPROCHO vs performance factor correlation showed a Pearson R^2 ^of 0.54, suggesting that 54% of the observed difference in power output performance between CHO and NpPROCHO can be explained by differences in athletic performance level. Indeed, when the cyclists were divided into two equally sized groups based on their individually calculated performance factor (Table 1), ingestion of NpPROCHO resulted in improved power output-performance relative to ingestion of CHO in the lesser performing cyclists compared to the superior performing cyclists (-2.4% vs -1.9%, P < 0.05) (Figure 4). As for ingestion of PROCHO, no such effect was observed. Adding to this, in the lesser trained athletes, ingestion of NpPROCHO had a positive effect on power output performance relative to CHO compared to ingestion of PROCHO (ES = 1.08). This classifies as a *large *ES and signifies that the mean of the performance of the NpPROCHO group lies at the 88 percentile of the PROCHO group.

## Discussion

This is the first study to compare the effects of ingesting supplements of protein and hydrolyzed protein on physical endurance performance. The results show that, with the current protocol, there was no mean effect on 5-min mean-power performance of ingesting the marine hydrolyzed protein-supplement Nutripeptin™ (Np) together with protein and carbohydrate during the preceding 120 min of submaximal cycling. Importantly, however, ingestion of the NpPROCHO-beverage resulted in an interesting correlation between performance in the 5-min mean-power test and athletic performance level measured as a performance factor calculated from W_max_, VO_2max _and familiarization test 5-min mean-power performance. Although there are unavoidable uncertainties associated with analyzing data from a limited number of biological replicates, the confidence interval analysis suggested a high level of credibility. The data thus indicates that for cyclists with a lower performance level, herein those showing VO_2max _values in the lower part of the participant cohort (decreasing towards 60 ml·kg^-1^·min^-1^), the Np-supplement may have had an ergogenic effect on 5-min mean-power performance compared to CHO alone. Indeed, when the cyclists were divided into two equally sized groups based on athletic performance level, NpPROCHO improved 5-min mean-power output-performance relative to CHO in the lesser performing athletes but not in the superior performing athletes. The ergogenic effect in the lesser performing cyclists was associated with a large effect size. This brings forward a hypothesized delay in skeletal muscle fatigue, which could have to do with modulation of cellular events such as depletion of glycogen levels, removal of waste products or oxidative ATP production. In addition to this, the data suggests that ingestion of unprocessed protein together with carbohydrate during 120 min of submaximal cycling does not improve performance in a subsequent 5-min mean-power test compared to ingestion of carbohydrate alone. This is in line with results from several other studies [2,5,6].

All three beverages investigated in this study contained carbohydrate levels corresponding to intake of 60 g·h^-1^. This should have ensured maximal rates of exogeous carbohydrate oxidation [1]. In each of the two beverages containing protein, the protein fraction corresponded to an intake of about 15 g·h^-1^, increasing the overall caloric content of these beverages. Accordingly, the apparent lack of an ergogenic effect of supplying an iso-carbohydrate beverage with protein or hydrolyzed protein suggests that protein offers no acute caloric advantage for a performing athlete. In agreement with this, the three beverages were associated with similar RER values throughout the prolonged submaximal exercise, suggesting that protein ingestion did not result in a major metabolic shift towards amino acid oxidation or fatty acid. As for the Nutripeptin™-containing beverage, this lack of a metabolic shift contrasts the hypothesized role of the supplement as a signal that provides a switch towards fatty acids. Nevertheless, NpPROCHO ingestion but not PROCHO was associated with a possible ergogenic effect, despite the fact that the two beverages isoprotein-caloric. Notably, for both of the protein-containing beverages the ingested protein seemed to be absorbed and catabolized, as evaluated from the similar increases in blood concentrations of the protein-degradation by-product BUN measured subsequent to 120 min of steady-state cycling.

An interesting consequence of the correlative relation between NpPROCHO performance and athletic performance level was that the beverage resulted in lowered performance in the better athletes. As touched upon in the previous discussion this could be an effect of the specific protocol utilized in this study and the outcome may have been different if the pre-exhaustive cycling phase had been longer-lasting. These results are not easy to explain based on current knowledge, especially as the PROCHO beverage did not result in a similar correlation. A speculative explanation could be a potential difference in the insulinogenic response offered by the two beverages. Previous studies have at least shown that ingestion of hydrolyzed protein is associated with a substantially greater insulinogenic response than ingestion of intact protein [27,28]. Mechanistically, this response has been linked to hypoglycaemia, and has been linked to lowered physical performance during early phases of exercise [29]. On the other hand, an elevated insulinogenic response has also been associated with a quantitative increase in glycogen synthesis, which in turn is likely to lower glycogen turn-over rates [22] an thereby delay exhaustion of glycogen stores. This could explain the improved performance found in the lower performing atheletes while ingesting NpPROCHO.

The potential ergogenic effect of Nutripeptin™ on long-lasting physical performance is either related to its physical status (i.e. it consist of degraded protein) or to its chemical composition (i.e. the amino acid composition). As for the first explanation, Saunders et al. [10] speculated that hydrolyzed protein is absorbed more efficiently across the gastrointestinal (GI) wall than intact proteins and that this may mediate improved performance. This would result in a more rapid and larger increase in [protein/amino acids] in blood plasma, with potential physiological effects such as an augmented insulinogenic response. In our opinion, this is unlikely to have been the case in our study, primarily because the similar increase in BUN values observed for the two protein beverages suggests that the performance-related differences between the beverages was not caused by differences in uptake or oxidation rates of amino acids. Secondarily, the ingestion of intact whey protein and hydrolyzed whey protein has been shown to be associated with similar absorption kinetics, with hydrolyzed protein actually being associated with slower insulinogenic kinetics [27]. As for the second potential explanation, regarding a role for the chemical composition of Nutripeptin™, this has previously been suggested to underly the increased oxidative capacity and loss of visceral fat observed in rats after long-term ingestion of hydrolyzed fish protein [19,20], suggesting a metabolic shift towards fatty acids. This, however, is unlikely to be the explanation behind the potential ergogenic effect of NPPROCHO ingestion relative to CHO, as the RER data suggests that similar substrate sources were utilized for ATP production for all three beverage treatments.

## Conclusions

In summary, our results gives support to the hypothesis that co-ingestion of carbohydrate and unprocessed protein does not improve 5 min mean-power performance following 120-min prolonged submaximal cycling compared to ingestion of CHO alone. Correlational analysis indicate that Np added with whey protein and carbohydrate may provide ergogenic benefit for lesser trained athletes. However, the current data precludes us from definitively positing this, and mechanisms of such possible effects remain unknown. The effect seems to be restricted to athletes that were approaching their limits of physical achievement. To further elucidate this intriguing prospect, future research should focus on protocols with longer-lasting pre-exhaustive submaximal exercise (> 120 min), followed by a time trial, ensuring a more competition-like simulation for cyclists. Future studies should also include surveillance of parameters such as insulinogenic responses and should address degrees of muscular exertion by measuring parameters such as glycogen content. For athletes competing in events such as cycling, ingestion of Nutripeptin™ could prove an essential step towards optimizing prolonged endurance performance.

## Abbreviations

CHO: Beverage containing carbohydrate; PROCHO: Beverage containing protein + carbohydrate; NpPROCHO: Beverage containing Nutripeptin™ + protein + carbohydrate.

## Competing interests

The authors have no professional relationship with companies or manufacturers who may benefit from the results of the present study. The authors' interpretation of the results does not constitute endorsement of the product. The study was partially funded by NutriMarine Life Science AS. In accordance with the authors' declared independency, NutriMarine Life Science AS was not at any point involved in study design, data sampling, data analysis or preparation of the written product.

## Authors' contributions

GV, BRR and SE contributed to conception and design, analysis and interpretation of data. SE drafted the paper and all authors contributed by revising it critically. All authors approved the final version to be published. The experiments were performed in the laboratory facility at Lillehammer University College.

## References

[B1] Jeukendrup AE Carbohydrate intake during exercise and performance Nutrition 2004 20 669 677 10.1016/j.nut.2004.04.017 15212750

[B2] Van Essen M Gibala MJ Failure of Protein to Improve Time Trial Performance when Added to a Sports Drink Med Sci Sports Exerc 2006 38 1476 1483 10.1249/01.mss.0000228958.82968.0a 16888462

[B3] Stearns RL Emmanuel H Volek JS Casa DJ Effects of Ingesting Protein in Combination With Carbohydrate During Exercise on Endurance Performance: A Systematic Review With Meta-Analysis J Strength Condit Res 2010 24 2192 2202 10.1519/JSC.0b013e3181ddfacf 20683237

[B4] Ivy JL Res PT Sprague RC Widzer MO Effect of a carbohydrate-protein supplement on endurance performance during exercise of varying intensity Int J Sport Nutr Exerc Metab 2003 13 382 395 14669937 10.1123/ijsnem.13.3.382

[B5] Osterberg KL Zachwieja JJ Smith JW Carbohydrate and carbohydrate + protein for cycling time-trial performance J Sports Sci 2008 26 227 233 10.1080/02640410701459730 18074296

[B6] Breen L Tipton KD Jeukendrup AE No Effect of Carbohydrate-Protein on Cycling Performance and Indices of Recovery Med Sci Sports Exerc 2010 42 1140 1148 19997018 10.1249/MSS.0b013e3181c91f1a

[B7] Saunders MJ Kane MD Todd MK Effects of a Carbohydrate-Protein Beverage on Cycling Endurance and Muscle Damage Med Sci Sports Exerc 2004 36 1233 1238 10.1249/01.MSS.0000132377.66177.9F 15235331

[B8] Toone RJ Betts JA Isocaloric Carbohydrate Versus Carbohydrate-Protein Ingestion and Cycling Time-Trial Performance Int J Sport Nutr Exerc Metab 2010 20 34 43 20190350 10.1123/ijsnem.20.1.34

[B9] Jeukendrup AE Tipton KD Gibala MJ Protein Plus Carbohydrate Does Not Enhance 60-km Time-Trial Performance Int J Sport Nutr Exerc Metab 2009 19 335 337 19827459 10.1123/ijsnem.19.4.335

[B10] Saunders MJ Moore RW Kies AK Luden ND Pratt CA Carbohydrate and Protein Hydrolysate Coingestion's Improvement of Late-Exercise Time-Trial Performance Int J Sport Nutr Exerc Metab 2009 19 136 149 19478339 10.1123/ijsnem.19.2.136

[B11] Saunders MJ Protein Plus Carbohydrate Does Not Enhance 60-km Time-Trial Performance Response Int J Sport Nutr Exerc Metab 2009 19 337 339 10.1123/ijsnem.19.4.335 19827459

[B12] Davidsen PK Gallagher IJ Hartman JW Tarnopolsky MA Dela F Helge JW Timmons JA Phillips SM High responders to resistance exercise training demonstrate differential regulation of skeletal muscle microRNA expression J Appl Physiol 2011 110 309 317 10.1152/japplphysiol.00901.2010 21030674

[B13] Timmons JA Variability in training-induced skeletal muscle adaptation J Appl Physiol 2011 110 846 853 10.1152/japplphysiol.00934.2010 21030666 PMC3069632

[B14] Timmons JA Knudsen S Rankinen T Koch LG Sarzynski MA Jensen T Keller P Scheele C Vollaard NB Nielsen S Using molecular classification to predict gains in maximal aerobic capacity following endurance exercise training in humans J Appl Physiol 2010 01295 02009 10.1152/japplphysiol.01295.2009 PMC2886694 20133430

[B15] Manninen AH Protein hydrolysates in sports nutrition Nutr Metabol 2009 6 38 10.1186/1743-7075-6-38 PMC2761917 19785737

[B16] Buckley JD Thomson RL Coates AM Howe PRC DeNichilo MO Rowney MK Supplementation with a whey protein hydrolysate enhances recovery of muscle force-generating capacity following eccentric exercise J Sci Med Sport/Sports Med Aust 2010 13 178 181 10.1016/j.jsams.2008.06.007 18768358

[B17] Beelen M Tieland M Gijsen AP Vandereyt H Kies AK Kuipers H Saris WHM Koopman R van Loon LJC Coingestion of Carbohydrate and Protein Hydrolysate Stimulates Muscle Protein Synthesis during Exercise in Young Men, with No Further Increase during Subsequent Overnight Recovery J Nutr 2008 138 2198 2204 10.3945/jn.108.092924 18936219

[B18] Boirie Y Dangin M Gachon P Vasson M-P Maubois J-L Beaufrère B Slow and fast dietary proteins differently modulate postprandial protein accretion Proc Natl Acad Sci USA 1997 94 14930 14935 10.1073/pnas.94.26.14930 9405716 PMC25140

[B19] Liaset B Madsen L Hao Q Criales G Mellgren G Marschall HU Hallenborg P Espe M Froyland L Kristiansen K Fish protein hydrolysate elevates plasma bile acids and reduces visceral adipose tissue mass in rats Biochim Biophys Acta Mol Cell Biol Lipids 2009 1791 254 262 10.1016/j.bbalip.2009.01.016 19416649

[B20] Liaset B Espe M Nutritional composition of soluble and insoluble fractions obtained by enzymatic hydrolysis of fish-raw materials Process Biochem 2008 43 42 48 10.1016/j.procbio.2007.10.007

[B21] Hermansen L Hultman E Saltin B Muscle Glycogen during Prolonged Severe Exercise Acta Physiol Scand 1967 71 129 139 10.1111/j.1748-1716.1967.tb03719.x 5584522

[B22] Sherman W Metabolism of sugars and physical performance Am J Clin Nutr 1995 62 228S 241S 7598080 10.1093/ajcn/62.1.228S

[B23] Ronnestad BR Hansen EA Raastad T Effect of heavy strength training on thigh muscle cross-sectional area, performance determinants, and performance in well-trained cyclists Eur J Appl Physiol 2010 108 965 975 10.1007/s00421-009-1307-z 19960350

[B24] Lukaski HC Vitamin and mineral status: Effects on physical performance Nutrition 2004 20 632 644 10.1016/j.nut.2004.04.001 15212745

[B25] Hansen E Jensen K Pedersen P Performance following prolonged sub-maximal cycling at optimal versus freely chosen pedal rate Eur J Appl Physiol 2006 98 227 233 10.1007/s00421-006-0266-x 16906415

[B26] Rønnestad BR Hansen EA Raastad T Strength training improves 5-min all-out performance following 185 min of cycling Scand J Med Sci Sports 2011 21 250 259 10.1111/j.1600-0838.2009.01035.x 19903319

[B27] Power O Hallihan A Jakeman P Human insulinotropic response to oral ingestion of native and hydrolysed whey protein Amino Acids 2009 37 333 339 10.1007/s00726-008-0156-0 18679613

[B28] Manninen AH Hyperinsulinaemia, hyperaminoacidaemia and post-exercise muscle anabolism: the search for the optimal recovery drink Br J Sports Med 2006 40 900 905 10.1136/bjsm.2006.030031 16950882 PMC2465040

[B29] Foster C Costill DL Fink WJ Effects of preexercise feedings on endurance performance Med Sci Sports Exerc 1979 11 1&hyhen 5 582616

